# Butyrate induced changes in Wnt-signaling specific gene expression in colorectal cancer cells

**DOI:** 10.1186/1756-0500-7-226

**Published:** 2014-04-09

**Authors:** Darina L Lazarova, Christopher Chiaro, Michael Bordonaro

**Affiliations:** 1 Department of Basic Sciences, The Commonwealth Medical College, 525 Pine Street, Scranton, PA 18509, USA

**Keywords:** Microarray, Colorectal cancer, Butyrate, Wnt, Gene expression

## Abstract

**Background:**

We have determined that butyrate, which is derived from the fermentation of dietary fiber in the colonic lumen, hyperactivates Wnt activity in colorectal (CRC) cells, and that this upregulation of Wnt signaling is causatively related to the induction of apoptosis. To better understand the genetic program regulated by butyrate-mediated Wnt hyperactivation, we performed total human genome microarray analyses on HCT-116 CRC cells in the presence or absence of a physiologically relevant concentration of butyrate. To evaluate changes in Wnt-specific gene expression, Wnt activity was suppressed with inducible dominant negative Tcf4 (DN-Tcf4). Six biological replicates of a full human genome microarray were performed, and the data deposited into the Gene Expression Omnibus database, according to Minimum Information About A Microarray Experiment standards.

**Results:**

Reporter assay and western blot data confirm that DN-Tcf4 is expressed at high levels in stably transfected HCT-116 cells upon cotreatment with doxycycline and butyrate, and that these cells exhibit a marked repression of butyrate-mediated Wnt hyperactivation. Analysis of six biological replicates of microarray analyses indicated that 1008 genes are modulated by butyrate (>two-fold, P < 0.01) in a Wnt signaling-specific manner, while 1587 genes are similarly modulated at P < 0.05. The modulated genes include members of a variety of gene families; including the Biological Process category, such as regulation of development, regulation of metabolism, cytokine and chemokine mediated signaling pathways, and DNA replication; the Cellular Component category such as cytoskeleton and organelle factors, and intermediate filaments; and the Molecular Function category, such as GTPase activator activity.

**Conclusions:**

We have identified, for the first time, in CRC cells, the total array of direct and indirect Wnt-target genes whose expression is modulated by butyrate. Knowledge of the molecular mechanisms determining the response of CRC cells to butyrate *in vitro* may assist in determining more effective preventive and therapeutic strategies against CRC.

## Background

Canonical Wnt signaling is induced by the binding of Wnt ligands to cell surface receptors, resulting in the inactivation of intact Axin 1-containing complexes [1], and accumulation of transcriptionally active beta-catenin [1-5] which interacts with Tcf DNA binding proteins [6-11]. Beta-catenin-Tcf transcriptional complexes drive transcription from Tcf site-containing promoters, including both reporter constructs and endogenous Wnt responsive genes containing Tcf/Lef sites [8,9] and refs. therein. Constitutively active Wnt signaling, caused by mutations in the *APC* and *beta-catenin* genes [8-10] promotes colonic cell proliferation and tumorigenesis; however, both relatively high and relatively low levels of Wnt transcriptional activity lead to CRC cell apoptosis [12-16].

Colorectal cancer (CRC) may to some extent be preventable through diet [13,14,17-22]. The protective action of dietary fiber against CRC has been attributed to its fermentation in the colon, producing the histone deacetylase inhibitor (HDACi) butyrate [23,24]. HDACis induce cell cycle arrest, differentiation, and/or apoptosis of CRC cells [25,26]; the HDACis butyrate and trichostatin A (TSA), which induce apoptosis in CRC cells *in vitro*, hyperactivate Wnt transcriptional activity in these cells [12-14]. The ability of butyrate, and other HDACis, to promote CRC apoptosis and repress cell growth, is casually related to the degree of Wnt hyperactivation induced by these agents [12-14].

To determine the genetic program regulated by butyrate-mediated Wnt hyperactivation, we performed total human microarray analysis on HCT-116 CRC cells. Cells were treated with a physiologically relevant concentration (5 mM) of butyrate [24] or left untreated. To evaluate changes in Wnt-specific gene expression, Wnt activity was suppressed with inducible dominant negative Tcf4 (DN-Tcf4), which competes with endogenous beta-catenin-Tcf (BCT) complexes for binding to promoter sequences of Wnt target genes [8]. We identified more than one thousand genes that exhibited Wnt-specific changes in expression (>two-fold, P < 0.01) after exposure to butyrate. These changes in gene expression include a variety of gene families, representing clusters of functionally related gene products.

Thus, in this short Research Article/Data Note, we identify, for the first time, in a human CRC cell line, how exposure to butyrate modulates the expression of genes that are direct or indirect targets of Wnt signaling.

## Methods

### Plasmids, cell lines, transfection, luciferase assay

pTOPFLASH (TOP), pFOPFLASH (FOP), and inducible DN-Tcf4 were from Dr. H. Clevers (UMC Utrecht, Utrecht, Netherlands). Tet repressor plasmid was from Invitrogen (Carlsbad, CA). HCT-116 cells were obtained from the American Type Culture Collection (ATCC). Transfection with lipofectamine 2000 and luciferase assays were performed as previously described [12-14]. Measurements of Wnt transcriptional activity were performed with the TOP/FOP luciferase reporter system that has wild-type (TOP) or mutant (FOP) Tcf binding sites upstream of a minimal c-fos promoter (8, 9).

### Nucleofection and stable transfection

Standard protocol was utilized according to manufacturer’s instructions. Setting D-32 was used to nucleofect HCT-116 cells. Routinely, 2 μg of DNA and 1 × 10^6^ cells were used per well (6 well plate) for each nucleofection. HCT-116 cells were stably transfected, via nucleofection, with a vector for Tet repressor and a Tet-inducible DN-Tcf4 vector [27,28]. Cells were selected with 100 μg/ml zeocin and 5 μg/ml blasticidin, and DN-Tcf4 expression was induced by doxycycline (4 μg/ml), followed by clonal selection. Clones were assayed based on doxycycline-inducible suppression of Wnt activity as measured by reporter assays, and expression of DN-Tcf4 as evaluated by western blot analysis.

### Genus biosystems total human genome microarray analysis

After treatment with or without 4 μg/ml doxycycline and then cotreatment with or without 5 mM sodium butyrate (NaB) for 17.5 hr, cells were washed with 1 × PBS, scraped into PBS and pelleted; the pellets were snap frozen in liquid nitrogen and sent to Genus Biosystems (Northbrook, IL) for RNA extraction and microarray analyses, utilizing the Agilent human whole genome oligo microarray. Briefly, RNA extraction and array analyses were performed by Genus as follows. RNA was extracted and purified with Ambion Ribopure isolation, with RNA quality assessed by an Agilent Bioanalyzer. Following first and second strand cDNA synthesis, cRNA target was prepared, fragmented to a uniform size, and hybridized to Agilent Human v2 GE 4x44K arrays. Slides were subsequently washed and scanned on an Agilent G2565 Microarray Analyzer and the resulting data were analyzed with Agilent Feature Extraction and GeneSpring GX v7.3.1 software. A total of six biological replicates of the experiment were performed.

### Western blotting

Anti-FLAG antibody (Origene, Rockville, MD) was used to detect the FLAG-tagged DN-Tcf4. Actin was used a loading control using anti-actin antibody (Sigma, St. Louis, MO). Protein isolation and western blotting were performed as previously described [12-14].

### Statistics

For gene transfection experiments, Students *t*-test was utilized, with statistical significance set at P < 0.05 for the Wnt reporter experiments. For microarray analyses, paired *T*-test was used at P < 0.01 and P < 0.05.

## Results and discussion

### Characterization of system

We have established that physiologically relevant concentrations of butyrate induce apoptosis, and repress clonal growth, in CRC cells dependent upon the hyperactivation of Wnt activity [12-14]. Therefore, it is likely that a set of Wnt signaling targeted genes mediates the high apoptotic response of CRC cells to butyrate. To better evaluate differences in gene expression that contribute to the butyrate response, we determined the pattern of gene expression changes due to butyrate treatment, focusing on genes which are direct or indirect Wnt targets. We identified these genes by microarray analyses of HCT-116 cells, in which Wnt signaling is suppressed. Thus, we established a stably transfected HCT-116 cell line expressing doxycycline-inducible DN-Tcf4. DN-Tcf4 is a form of Tcf4 that lacks the beta-catenin interaction domain and represses Wnt activity by competing with BCT complexes for access to DNA binding sites in Wnt target genes (8). We have previously shown that DN-Tcf4 efficiently represses the upregulation of Wnt activity by butyrate in a variety of CRC cell lines, including HCT-116 ([12-14] and data not shown).

**Figure 1:**
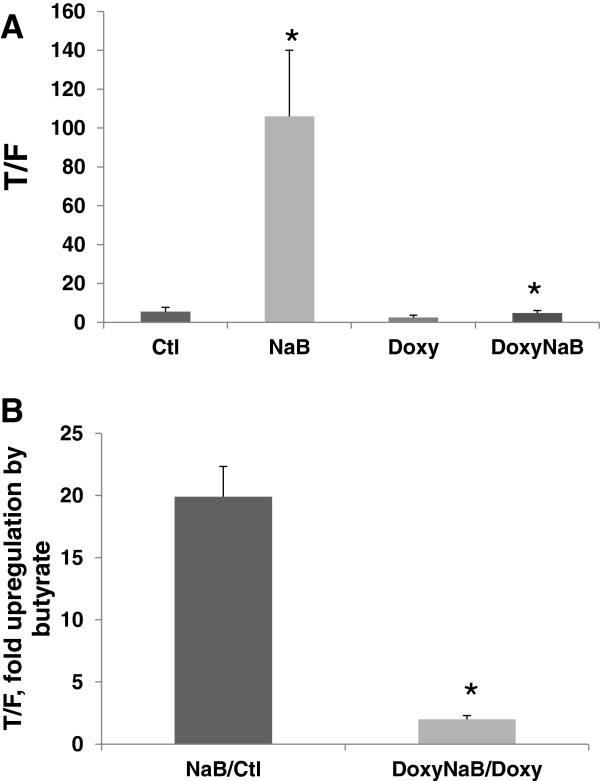
**Clone 19 exhibits doxycycline-induced downregulation of the Wnt hyperactivation by butyrate. (A)** Cells were transfected with TOPFlash or FOPFlash reporters and pRLTK (to control for transfection efficiency) and expression of DN-Tcf4 was induced by treatment with 4 μg/ml doxycycline; cells were also treated or not treated with 5 mM butyrate (NaB), both agents (DoxyNaB); control (Ctl) cells were not treated with either agent. After 17 hr of butyrate treatment, Wnt transcriptional activity was measured via luciferase assays. T/F represents the ratio of expression of TOPFlash (wild-type Wnt-responsive promoter) compared to FOPFlash (mutant promoter), measuring overall canonical Wnt activity. **(B)** Data from (A) represented by the fold-upregulation of Wnt activity in the presence (DoxyNaB/Doxy) or absence (NaB/Ctl) of doxycycline. (A) and (B) show data from four independent experiments. Bars, SDs. * = statistical significance.

In the stably transfected cell clone, induction with doxycycline repressed the ability of butyrate to upregulate Wnt activity, as expected (Figure 1A, B). Wnt activity was measured by luciferase reporter assays, comparing the expression from a Wnt-sensitive reporter (pTOPFLASH) to that of the control reporter that is not responsive to Wnt signaling (pFOPFLASH) ([12-14] and refs. therein). Untreated cells exhibited a TOP/FOP ratio, indicative of Wnt activity, of 5.5 which was increased to 106.0 (P < 0.002) by treatment with 5 mM butyrate. Treatment with doxycycline alone resulted in a TOP/FOP ratio of 2.5, which was increased to 4.7 with combinatorial treatment with both doxycycline and butyrate (P < 0.05). Thus, the fold-upregulation of Wnt activity by butyrate in the presence of doxycycline was approximately 10-fold lower (P < 0.001) than in the absence of doxycycline.

**Figure 2:**
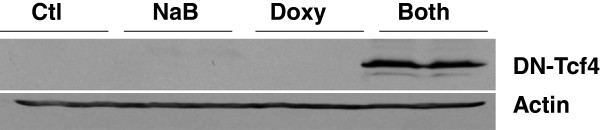
**Overexpression of DN-Tcf4 in induced HCT-116 cells.** A stably transfected clone of HCT-116 cells was treated with 4 μg/ml doxycycline (Doxy) and/or 5 mM NaB for 17.5 hr or left untreated. Protein was isolated, and DN-Tcf4 detected with anti-FLAG antibody in western blot analysis. Actin was analyzed as a control. Duplicate samples from a representative western blot shown.

Consistent with our previous findings with stable transfection of inducible DN-Tcf4 in DLD-1 CRC cells, and transient transfection of this vector in HCT-116 cells [13], basal expression of stably transfected DN-Tcf4 is minimal in the presence of doxycycline and absence of butyrate, but is at high levels in the presence of both agents (Figure 2). The likely reason for low basal levels of DN-Tcf4 in the absence of butyrate is counter-selection during clonal selection; clones that would express high levels of DN-Tcf4 in the presence of doxycycline and absence of butyrate would also likely express lower, but physiologically relevant, levels of DN-Tcf4 in the absence of doxycycline. This background expression would likely inhibit cell growth during clonal expansion compared to clones exhibiting lower DN-Tcf4 expression. Given the ability of butyrate to upregulate induced DN-Tcf4 expression in these cells, the relative repression of DN-Tcf4 expression in the absence of butyrate may involve **(a)** histone deacetylation of the DN-Tcf4 promoter sequences, which is reversed by the HDACi activity of butyrate; and/or **(b)** repressed expression of activating transcription factors or enhanced expression of transcriptional repressors, both targeting the DN-Tcf4 promoter. In the latter case, butyrate would be expected to favor DN-Tcf4 transcriptional activation through altered expression of these transcriptional factors.

Given that combinatorial treatment of cells with doxycycline and butyrate results in high expression of the Wnt inhibitor DN-Tcf4 (Figure 2), why does the combination of these two agents result in an increase in the TOP/FOP ratio compared to doxycycline alone (Figure 1A)? Butyrate increases the TOP/FOP ratio in CRC cells through the marked hyperactivation of Wnt signaling [12-14]. In the absence of DN-Tcf4 expression, the TOP/FOP ratio is increased from 5.5 to 106 by butyrate treatment (NaB vs. Ctl in Figure 1A). This extremely large activation of Wnt activity is not completely eliminated by the presence of DN-Tcf4. Thus, a modest 1.9-fold increase in the TOP/FOP ratio, from 2.5 to 4.7, is observed upon doxycycline/butyrate cotreatment compared to doxycycline alone (DoxyNaB vs. Doxy in Figure 1A).

However, the main experimental comparison for our microarray analyses was that of **(a)** butyrate treatment alone compared to control (NaB/Ctl) vs. **(b)** doxycycline and butyrate treatment compared to doxycycline alone (DoxyNaB/Doxy). Thus, the small upregulation of Wnt activity still induced by butyrate in the presence of DN-Tcf4 expression (1.9-fold) is an order of magnitude less (P < 0.001) than that induced by butyrate in the absence of DN-Tcf4 (19.3-fold) (compare NaB/Ctl to DoxyNaB/Doxy in Figure 1B). This 10-fold difference in Wnt hyperactivation by butyrate observed +/− DN-Tcf4 allows us to identify changes in Wnt-target gene expression induced by butyrate.

The observed expression pattern of DN-Tcf4 (Figure 2) is favorable, since the objective was to compare gene expression in the presence or absence of butyrate-enhanced Wnt signaling. Thus, we aimed at repressed Wnt activity upon butyrate treatment; this aim was achieved, as demonstrated by the markedly reduced Wnt hyperactivation shown in Figure 1.

We expected that known Wnt activity-targeted genes would exhibit reduced gene expression in the presence of induced DN-Tcf4, and this was confirmed. Table 1 shows the relative expression of selected Wnt/Tcf targeted genes [27-29]; some of these genes were previously shown to exhibit repressed expression in the presence of induced DN-Tcf4 in LS174T and DLD-1 CRC cells [27,28]. Relative expression levels are shown, after induction with doxycycline alone (Doxy) compared to untreated control (Ctl), or after induction and butyrate treatment (Doxy + NaB) compared to butyrate treatment alone (NaB). Induction with doxycycline alone resulted in moderate to no change in the expression of these genes, while the combination of doxycycline induction and butyrate treatment resulted in statistically significant downregulation. Therefore, as expected, expression of Wnt activity-targeted genes is downregulated upon the high induction of DN-Tcf4 observed after treatment of cells with doxycycline and butyrate.

**Table 1:** Relative expression of Wnt/Tcf-targeted genes upon doxycycline induction in the presence or absence of butyrate

**Relative expression of Wnt/Tcf-targeted genes**		
**Name**	**Doxy/Control**	**Doxy + NaB/NaB**
Axin2	*0.66*	*0.17*
BMP4	*0.79*	*0.40*
C-Myc	1.05	*0.35*
Cyclin D1	1.03	*0.50*
Dkk1	0.88	*0.33*
KITLG	0.91	*0.44*
Sox2	0.78	*0.62*
Sox4	0.93	*0.65*
Sox9	*1.21*	*0.60*
SP5	0.86	*0.29*
Vimentin	1.18	*0.71*

Numbers in italics represent differences that are statistically significant at P < 0.01.

### Experimental approach

The experimental approach was to use human whole genome microarray analyses to first identify all genes whose expression was up- or downregulated by butyrate, in a statistically significant manner, by > two-fold, in the absence of induced DN-Tcf4. We then repeated the analysis in the presence of doxycycline/butyrate-induced DN-Tcf4, and considered those genes whose expression was modulated by butyrate in the absence of doxycycline (intact Wnt signaling) but not similarly modulated in the presence of doxycycline (repressed Wnt signaling). Thus, we were interested in identifying those genes that are differentially expressed (>two-fold, P < 0.01) in the NaB vs. Ctrl comparison but not differentially expressed (>two-fold, P < 0.01) in the Doxy + NaB vs. Doxy comparison. The NaB vs. Ctrl comparison yields all genes differentially expressed after exposure to 5 mM butyrate, while the Doxy + NaB vs. Doxy comparison yields all butyrate-modulated genes except those that are Wnt activity-targeted, since doxycycline induction combined with butyrate treatment efficiently induces DN-Tcf4 expression (Figure 2). Additional information about the experimental design, samples, and raw data are included in Additional files 1 and 2.

### Identification of genes, the expression of which is modulated by butyrate-mediated Wnt signaling

**Figure 3:**
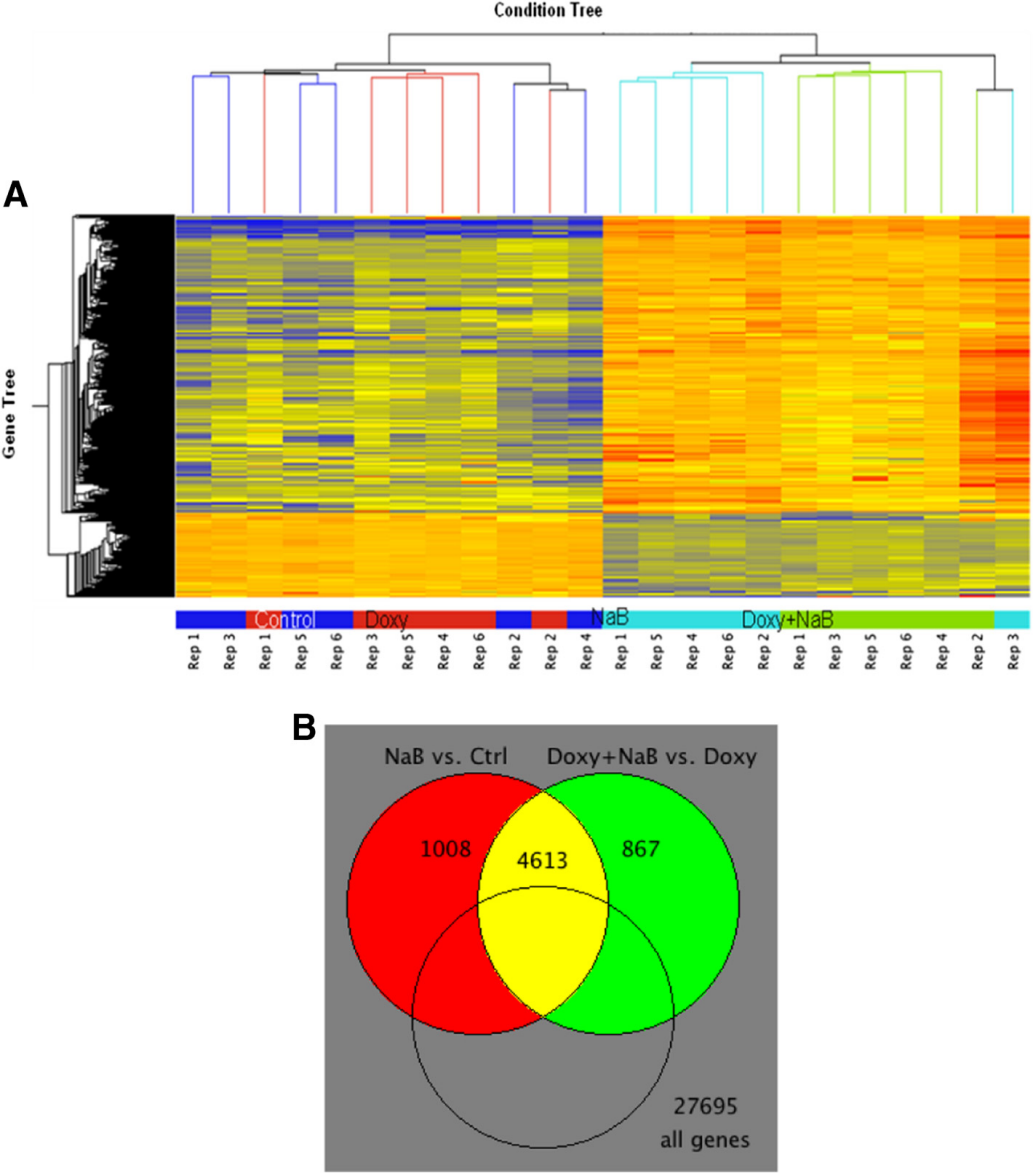
**Differential expression of genes in NaB vs. Control NOT Doxy + NaB vs. Doxy. (A)** Condition tree. Genes are displayed as normalized to the median expression across 4 samples within each experiment (>two-fold, paired *T*-test, P < 0.01, 1008 probes). Red/Orange = Up-regulated relative to median, Yellow = Median expression, Blue = Dn-regulated relative to median. **(B)** Venn diagram emphasizes comparison of NaB vs. Ctrl or Doxy + NaB vs. Doxy (>two-fold, paired *T*-test, P < 0.01).

Microarray analyses identified a number of genes whose expression levels are modified by butyrate in a manner sensitive to repression of Wnt activity by DN-Tcf4 [30]. Thus, a total of 1008 genes were identified which were modulated by butyrate alone (up- or downregulation) by at least 2-fold with P < 0.01, and 1587 genes at P < 0.05, but were not similarly modulated by butyrate when Wnt activity was repressed (induction of DN-Tcf4 by doxycycline and butyrate) (Figure 3).

Gene ontology analysis of the microarray data reveals a number of functionally relevant gene families, expression of which is influenced by butyrate in a Wnt signaling-dependent manner (Table 2). These gene families include the **(a)** Biological Process category, such as regulation of development, regulation of metabolism, cytokine and chemokine mediated signaling pathways, and DNA replication; **(b)** Cellular Component category such as cytoskeleton and organelle factors, and intermediate filaments; and **(c)** Molecular Function category, such as GTPase activator activity.

**Table 2:** Microarray data gene ontology classifications

**Gene ontology**		
**Microarray data gene ontology classifications**		
**Category** = the name of the category within the ontology.		
**Genes in category** = the total number of genes in the genome that have been assigned to the category		
**Genes in list in category** = the total number of genes both in the selected gene list and in the category.		
**Biological process**		
**Category**	**Genes in category**	**Genes in list in category**
GO:50769: positive regulation of neurogenesis	101	14
GO:51094: positive regulation of development	420	30
GO:50793: regulation of development	848	49
GO:45598: regulation of fat cell differentiation	59	9
GO:50772: positive regulation of axonogenesis	47	8
GO:1779: natural killer cell differentiation	4	3
GO:9991: response to extracellular stimulus	238	19
GO:50767: regulation of neurogenesis	280	21
GO:45600: positive regulation of fat cell differentiation	20	5
GO:45444: fat cell differentiation	126	12
GO:30844: positive regulation of intermediate filament depolymerization	2	2
GO:45108: regulation of intermediate filament polymerization and/or depolymerization	2	2
GO:30842: regulation of intermediate filament depolymerization	2	2
GO:45106: intermediate filament depolymerization	2	2
GO:45105: intermediate filament polymerization and/or depolymerization	2	2
GO:48541: Peyer’s patch development	7	3
GO:50789: regulation of biological process	8048	292
GO:31667: response to nutrient levels	211	16
GO:1764: neuron migration	85	9
GO:48537: mucosal-associated lymphoid tissue development	8	3
GO:9892: negative regulation of metabolism	1238	58
GO:45595: regulation of cell differentiation	612	33
GO:51093: negative regulation of development	292	19
GO:6066: alcohol metabolism	576	31
GO:31324: negative regulation of cellular metabolism	1126	52
GO:31668: cellular response to extracellular stimulus	64	7
GO:30901: midbrain development	34	5
GO:48715: negative regulation of oligodendrocyte differentiation	11	3
GO:9912: auditory receptor cell fate commitment	4	2
GO:31670: cellular response to nutrient	4	2
GO:19221: cytokine and chemokine mediated signaling pathway	249	16
GO:42423: catecholamine biosynthesis	24	4
GO:1709: cell fate determination	38	5
GO:6260: DNA replication	343	20
GO:8089: anterograde axon cargo transport	13	3
GO:50768: negative regulation of neurogenesis	89	8
GO:30879: mammary gland development	39	5
GO:45773: positive regulation of axon extension	25	4
GO:45686: negative regulation of glial cell differentiation	25	4
GO:17085: response to insecticide	25	4
GO:50770: regulation of axonogenesis	91	8
GO:15879: carnitine transport	14	3
GO:19800: peptide cross-linking via chondroitin 4-sulfate glycosaminoglycan	5	2
GO:48732: gland development	113	9
**Cellular component**		
**Category**	**Genes in category**	**Genes in list in category**
GO:5622: intracellular	16495	558
GO:43229: intracellular organelle	13641	467
GO:43226: organelle	13647	467
GO:797: condensin core heterodimer	2	2
GO:43232: intracellular non-membrane-bound organelle	4379	168
GO:43228: non-membrane-bound organelle	4379	168
GO:796: condensin complex	9	3
GO:5856: cytoskeleton	2219	92
GO:15630: microtubule cytoskeleton	1021	48
GO:5813: centrosome	467	26
GO:118: histone deacetylase complex	72	7
GO:43231: intracellular membrane-bound organelle	12378	415
GO:43227: membrane-bound organelle	12386	415
GO:16272: prefoldin complex	14	3
GO:31618: nuclear centric heterochromatin	5	2
GO:5882: intermediate filament	223	14
**Molecular function**		
**Category**	**Genes in category**	**Genes in list in category**
GO:5096: GTPase activator activity	397	25
GO:8118: N-acetyllactosaminide alpha-2,3-sialyltransferase activity	3	2
GO:16861: intramolecular oxidoreductase activity, interconverting aldoses and ketoses	11	3
GO:4873: asialoglycoprotein receptor activity	4	2
GO:8307: structural constituent of muscle	70	7
GO:8047: enzyme activator activity	573	30
GO:19894: kinesin binding	25	4
GO:31730: CCR5 chemokine receptor binding	5	2
GO:17123: Ral GTPase activator activity	5	2

Clusters of genes whose expression is modulated as described in Figure 3 and Table 1, grouped into functionally relevant categories.

Our results are most consistent with the modulation of multiple physiological and cell signaling pathways by butyrate-hyperinduced Wnt activity in CRC cells. These data can be utilized to identify relevant gene targets whose expression can be up- or down-regulated as part of preventive and/or therapeutic approaches against CRC. Further, the array findings can be utilized to better understand the mechanisms behind the physiological effects of butyrate on neoplastic colonic cells, particularly those effects dependent upon modulation of Wnt signaling. In addition, an understanding of Wnt-dependent butyrate-mediated changes in gene expression can inform about the development of butyrate resistance in CRC, which we have shown involves repressed induction of Wnt signaling in the presence of butyrate [14]. Therefore, future studies will be aimed at **(a)** up- or down-regulating relevant genes *in vitro* using overexpression or knockdown strategies, to ascertain effects on cell physiology, including, but not limited to, cell growth, differentiation, and apoptosis, as well as response to butyrate and other HDACis; and **(b)***in vivo* targeted gene overexpression or knockout in murine models of CRC, to evaluate effects on intestinal morphology, tumor formation, and response to dietary or therapeutic interventions, utilizing fiber/butyrate or other HDACis that mimic the effects of butyrate in cell culture [14]. These additional findings can also contribute to the development of more efficacious anti-CRC preventive/therapeutic methodologies.

## Conclusions

In summary, this study is the first examination of the total set of direct and indirect Wnt-target genes whose expression is modulated by butyrate in a human CRC cell line. These data suggest a large number of potential gene targets for anti-CRC therapeutic intervention, particularly for those approaches involving fiber/butyrate/HDACis. Knowledge of the molecular mechanisms determining the response of CRC cells to butyrate *in vitro* may assist in determining more effective preventive and therapeutic strategies against CRC.

### Availability of supporting data

The data set supporting the results of this article is available in the Gene Expression Omnibus (GEO) repository at http://www.ncbi.nlm.nih.gov/geo/query/acc.cgi?acc=GSE54127. Data are according to Minimum Information About A Microarray Experiment (MIAME) standards.

## Abbreviations

CRC: Colorectal cancer; BCT: Beta-catenin-Tcf; HDACi: Histone deacetylase inhibitor; DN-Tcf4: Dominant-negative Tcf4; NaB: Sodium butyrate; Doxy: Doxycycline; GEO: Gene Expression Omnibus; MIAME: Minimum information about a microarray experiment; TOP: pTOPFLASH expression activity; FOP: pFOPLASH reporter activity.

## Competing interests

The authors declare that they have no competing interests.

## Authors’ contributions

DL helped conceive the study, contributed to the cell culture work, and assisted in the drafting of the manuscript. CC isolated the appropriate stably transfected clone, performed the reporter assays and western blotting required to characterize the clone. MB conceived of the study, designed the experimental approach and oversaw all phases of the project, performed some of the transfections required to produce the stably transfected lines, interpreted the data, and drafted the manuscript. All authors read and approved the final manuscript.

## Supplementary Material

Additional file 1: **Experimental Overview, part 1.** Description Contains an overview of the microarray experiment for the first biological replicate of samples. Click here for file

Additional file 2: **Experimental Overview, part 2.** Description; Contains an overview of the microarray experiment for the last five biological replicates of samples. Click here for file

## References

[B1] Li VSW Ng SS Boersema PJ Low TY Karthaus WR Gerlach JP Mohammed S Heck AJR Maurice MM Mahmoudi T Clevers H Wnt signaling through inhibition of β-catenin degradation in an intact Axin1 complex Cell 2012 149 1245 1256 10.1016/j.cell.2012.05.002 22682247

[B2] Cook D Fry M Hughes K Sumathipala R Woodgett J Dale T Wingless inactivates glycogen synthase kinase-3 via an intracellular signaling pathway which involves a protein kinase C EMBO J 1996 15 4526 4536 8887544 PMC452182

[B3] Rubinfeld B Souza B Albert I Muller O Chamberlain SH Masiarz FR Munimetsu S Polakis P Association of the APC gene product with β-catenin Science 1993 262 1731 1734 10.1126/science.8259518 8259518

[B4] Su L-K Vogelstein B Kinzler KW Association of the APC tumor suppresser protein with catenins Science 1993 262 1734 1737 10.1126/science.8259519 8259519

[B5] Munemitsu S Albert I Souza B Rubinfeld B Polakis P Regulation of intracellular β-catenin levels by the adenomatous polyposis coli (APC) tumor-suppressor protein Proc Natl Acad Sci U S A 1995 92 3046 3050 10.1073/pnas.92.7.3046 7708772 PMC42356

[B6] Behrens J Von Kries JP Kuhl M Bruhn L Wedlich D Grosschedl R Birchmeier W Functional interaction of β-catenin with the transcriptional factor LEF-1 Nature 1996 382 638 642 10.1038/382638a0 8757136

[B7] Molenaar M Van De Wetering M Osterwegel M XTcf-3 transcription factor mediates β-catenin-induced axis formation in Xenopus embryos Cell 1996 86 391 399 10.1016/S0092-8674(00)80112-9 8756721

[B8] Korinek V Barker N Morin PJ Van Wichen D De Weger R Kinzler KW Vogelstein B Clevers H Constitutive transcriptional activation by a beta-catenin-Tcf complex in APC−/− colon carcinoma Science 1997 275 1784 1787 10.1126/science.275.5307.1784 9065401

[B9] Morin J Sparks AB Korinek V Barker N Clevers H Vogelstein B Kinzler KW Activation of beta-catenin-Tcf signaling in colon cancer by mutations in beta-catenin or APC Science 1997 275 1787 1790 10.1126/science.275.5307.1787 9065402

[B10] Bienz M Clevers H Linking colorectal cancer to Wnt signaling Cell 2000 103 311 320 10.1016/S0092-8674(00)00122-7 11057903

[B11] Roose J Clevers H Tcf transcription factors: molecular switches in carcinogenesis Biochim Biophys Acta 1999 87456 M23 M27 10528152 10.1016/s0304-419x(99)00026-8

[B12] Bordonaro M Mariadason JM Aslam F Heerdt BG Augenlicht LH Butyrate-induced apoptotic cascade in colonic carcinoma cells: modulation of the beta-catenin-Tcf pathway and concordance with effects of sulindac and trichostatin A but not curcumin Cell Growth Differ 1999 10 713 720 10547075

[B13] Lazarova DL Bordonaro M Carbone R Sartorelli AC Linear relationship between WNT activity levels and apoptosis in colorectal carcinoma cells exposed to butyrate Internat J Cancer 2004 110 523 531 10.1002/ijc.20152 15122584

[B14] Bordonaro M Lazarova DL Sartorelli AC The activation of beta-catenin by Wnt signaling mediates the effects of histone deacetylase inhibitors Exp Cell Res 2007 313 1652 1666 10.1016/j.yexcr.2007.02.008 17359971 PMC3919021

[B15] Albuquerque C Breukel C van der Luijt R Fidalgo P Lage P Slors FGM Leitao CN Fodde R Smits R The just-right signaling model: APC somatic mutations are selected based on a special level of activation of the beta-catenin signaling cascade Hum Mol Genet 2002 11 1549 1560 10.1093/hmg/11.13.1549 12045208

[B16] Bordonaro M Lazarova D Sartorelli AC Hyperinduction of Wnt signaling: a new paradigm for the treatment of colorectal cancer? Oncol Res 2008 17 1 9 18488710 10.3727/096504008784046108

[B17] Richter M Jurek D Wrba F Kaserer K Wurzer G Karner-Hanusch J Marian B Cells obtained from colorectal microadenomas mirror early premalignant growth patterns *in vitro* Euro J Cancer 2002 38 1937 1945 10.1016/S0959-8049(02)00158-2 12204677

[B18] Kautenberger T Beyer-Sehlmeyer G Festag G Haag N Kuhler S Kuchler A Weise A Marian B Peters WHM Liehr T Claussen U Pool-Zobel BL The gut fermentation product butyrate, a chemopreventive agent, suppresses glutathione S-transferase theta (hGSTT1) and cell growth more in human colon adenoma cells (LT97) than tumor (HT29) cells J Cancer Res Clin Oncol 2005 131 692 700 10.1007/s00432-005-0013-4 16133571 PMC12161184

[B19] Brabletz T Jung A Spaderna S Hlubek F Kirchner T Migrating cancer stem cells – an integrated concept of malignant tumour progression Nat Rev 2005 5 744 749 10.1038/nrc1694 16148886

[B20] Bingham SA Day NE Luben R Ferrari P Slimani N Norat T Clavel-Chapelon F Kesse E Nieters A Boeing H Tjonneland A Overvad K Martinzez C Dorronsoro M Gonzalez CA Key TJ Trichopolou A Naska A Vineis P Tumino R Krogh V Bueno-de-Mesquita HB Peeters PH Berglund G Hallmans G Lund E Skeie G Kaaks R Riboli E Dietary fibre in food and protection against colorectal cancer in the European Prospective Investigation into Cancer and Nutrition (EPIC): an observational study Lancet 2003 361 1496 1501 10.1016/S0140-6736(03)13174-1 12737858

[B21] Peters U Sinha R Chaterjee N Subar AF Ziegler RG Kuldorff M Bresalier R Weissfeld JL Flood A Schtzkin A Hayes RB Dietary fibre and colorectal adenoma in a colorectal cancer early detection programme Lancet 2003 361 1491 1495 10.1016/S0140-6736(03)13173-X 12737857

[B22] Bingham SA Norat T Moskal A Ferrari P Slimani N Chavel-Chapelon F Kesse E Nieters A Boeing H Tjonneland A Overvad K Martinzez C Dorronsoro M Gonzalez CA Ardanaz E Navarro C Quiros JR Key TJ Day NE Trichopoulo A Naska A Krogh V Tumino R Palli D Panico S Vineis P Bueno-de-Mesquita HB Ocke MC Peeters PHM Berglund G Is the association with fiber from foods in colorectal cancer confounded by folate intake? Cancer Epid Biom Prev 2005 14 1552 1556 10.1158/1055-9965.EPI-04-0891 15941971

[B23] Bingham S Mechanisms and experimental evidence relating dietary fibre and starch to protection against large bowel cancer Proc Nutr Soc 1990 49 153 171 10.1079/PNS19900021 2172992

[B24] Cummings JH Pomare EW Branch WJ Naylor CPE MacFarlane GT Short chain fatty acids in human large intestine, portal, hepatic, and venous blood Gut 1987 28 1221 1227 10.1136/gut.28.10.1221 3678950 PMC1433442

[B25] Heerdt BG Houston MA Augenlicht LH Potentiation by specific short-chain fatty acids of differentiation and apoptosis in human colonic carcinoma cell lines Cancer Res 1994 54 3288 3294 8205551

[B26] Medina V Young GP Edmonds B James R Appleton S Zalewski PD Induction of caspase-3 protease activity and apoptosis by butyrate and another inhibitor of histone deacetylase: dependence on protein synthesis and synergy with a mitochondrial/cytochrome c-dependent pathway Cancer Res 1997 57 3697 3707 9288776

[B27] Van de Wetering M Sancho E Verwij C de Lau W Oving I Hurlstone A van der Horn K Batlle E Coudreuse D Haramis A-P Tjon-Pon-Fong M Moerer P Van den Born M Soete G Pals S Eilers M Medema R Clevers H The β-catenin/TCF-4 complex imposes a crypt progenitor phenotype on colorectal cancer cells Cell 2002 111 241 250 10.1016/S0092-8674(02)01014-0 12408868

[B28] Van der Flier LG Sabates-Bellver J Oving I Haegebarth A De Palo M Anto M Van Gijn ME Suijkerbuijk S Van de Wetering M Marra G Clevers H The intestinal Wnt/TCF signature Gastroenterol 2007 132 628 632 10.1053/j.gastro.2006.08.039 17320548

[B29] The Wnt Homepage, Wnt target genes http://www.stanford.edu/group/nusselab/cgi-bin/wnt/target_genes

[B30] Gene Expression Omnibus (GEO) database http://www.ncbi.nlm.nih.gov/geo/query/acc.cgi?acc=GSE54127

