# Supplementary material for: Butyrate induced changes in Wnt-signaling specific gene expression in colorectal cancer cells
Source: BMC Res Notes. 2014 Apr 9;7:226. doi: 10.1186/1756-0500-7-226 (PMC4022274; doi:10.1186/1756-0500-7-226)
Supplement: Additional file 1: — Experimental Overview, part 1. Description Contains an overview of the microarray experiment for the first biological replicate of samples. [file 1756-0500-7-226-S1.pdf]

Microarray data for
The Commonwealth Medical College

Michael Bordonaro, Ph.D.

November 15, 2011

4 Human cell samples

Summary of procedures for microarray processing

RNA was extracted and purified using Ribopure (Ambion) RNA isolation

Total RNA samples were quantitated by UV spectrophotometry (OD260/280)

Quality of Total RNA was assessed using an Agilent Bioanalyzer

First and second strand cDNA was prepared from the total RNA samples

cRNA target was prepared from the DNA template and verified on the Bioanalyzer

cRNA was fragmented to uniform size and hybridized to Agilent Human v2 GE 4x44K arrays

Slides were washed and scanned on an Agilent G2565 Microarray Scanner

Data was analyzed with Agilent Feature Extraction and GeneSpring GX v7.3.1 software packages

Total RNA quality analysis

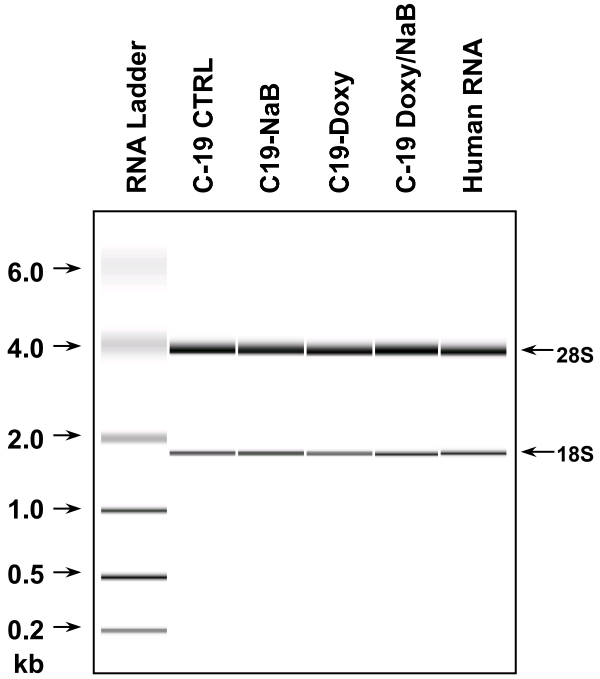

cRNA quality analysis

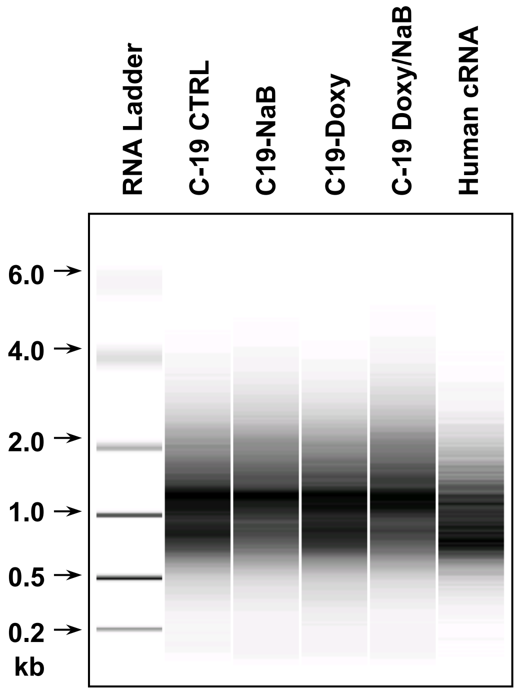

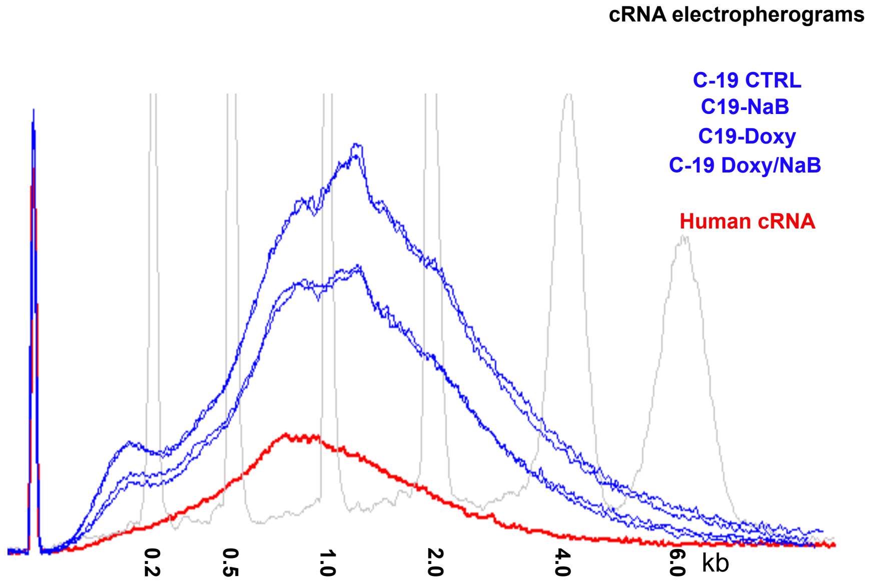

Scatterplot analysis

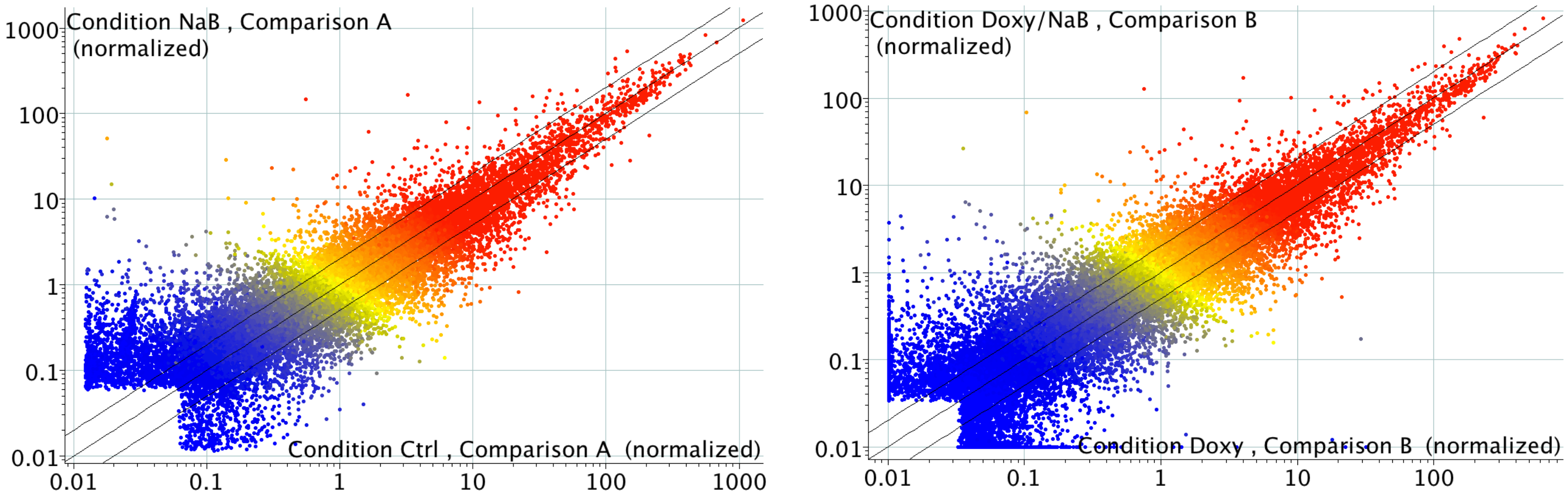

Probes above background in at least one sample per comparison (L:18,885 probes; R:22,169 probes). Intensity values are normalized to the 75th percentile intensity of each array. Diagonal lines indicate 2-fold differential expression. Red/Orange=High expression, Yellow=Medium expression, Blue=Low expression

Differentially Expressed Genes

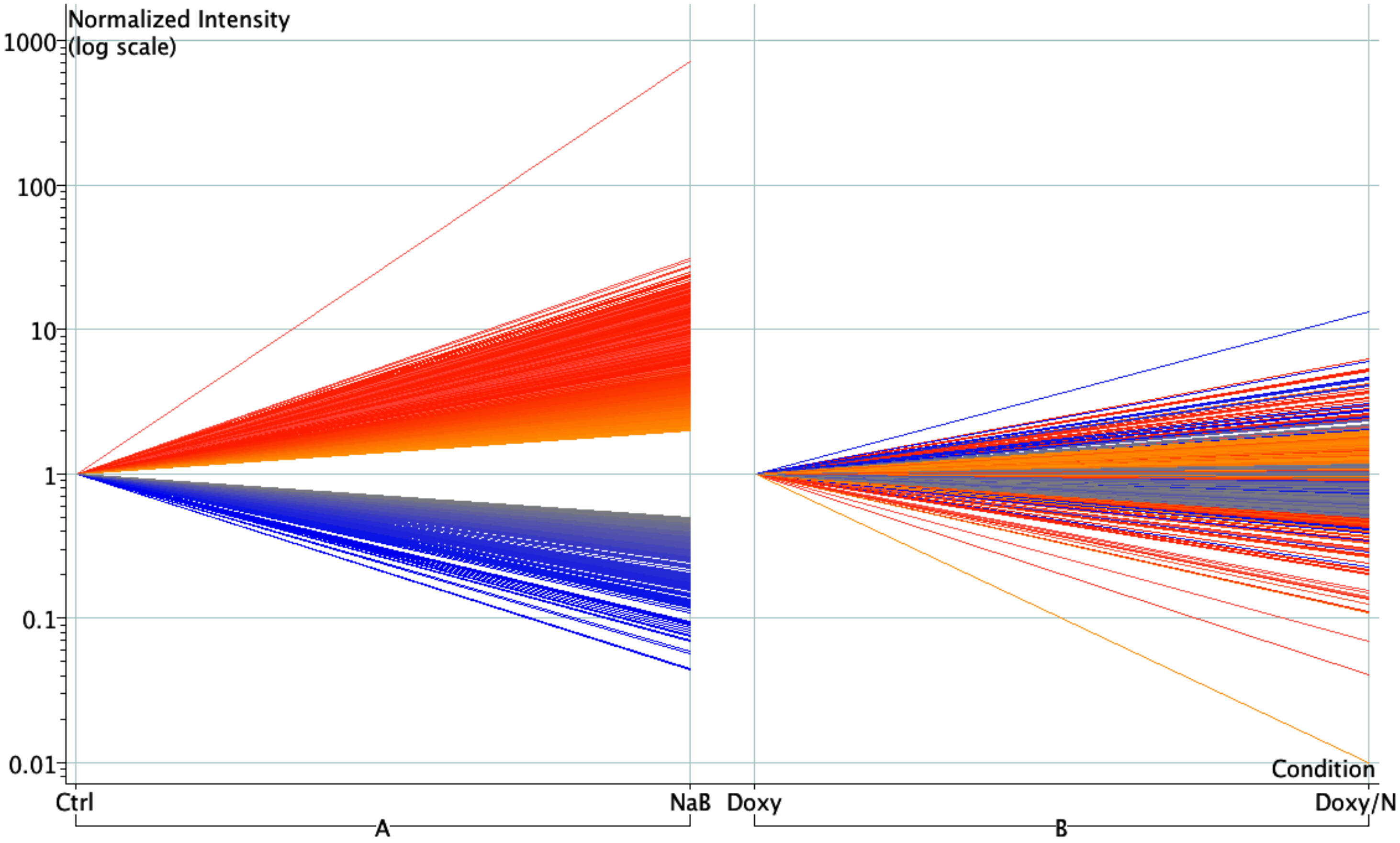

Differentially expressed (>2Fold) genes in NaB vs. Ctrl with opposite or undifferentiated expression in Doxy/NaB vs. Doxy (1,982 probes). Intensity values are normalized to the 75th percentile intensity of each array and further normalized to the control sample for each comparison. Genes are colored by the expression values in the NaB sample. Red/Orange=Up-regulated relative to Ctrl, Blue/Grey=Dn-regulated relative to Ctrl.

Condition and Gene tree clustering

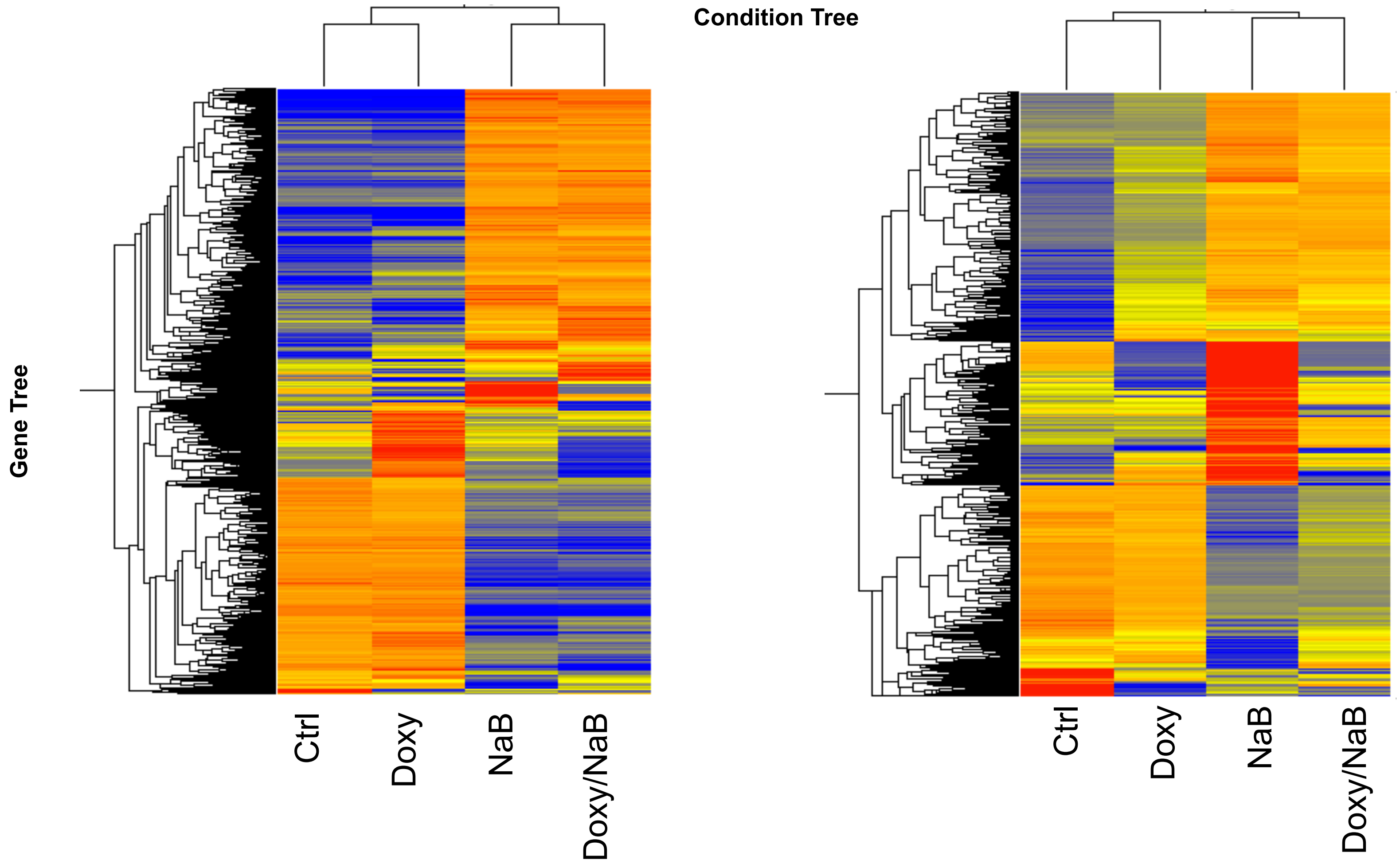

L: Differentially expressed genes (>2Fold, 10,963 probes) in at least one of two comparisons.
R: Differentially expressed genes (>2Fold, 1,982 probes) in NaB vs. Ctrl with opposite or undifferentiated expression in Doxy/NaB vs. Doxy.
All genes are normalized to the median expression across 4 samples. Red/Orange=Up-regulated relative to median, Yellow=Median expression, Blue=Dn-regulated relative to median.
