# Supplementary material for: Butyrate induced changes in Wnt-signaling specific gene expression in colorectal cancer cells
Source: BMC Res Notes. 2014 Apr 9;7:226. doi: 10.1186/1756-0500-7-226 (PMC4022274; doi:10.1186/1756-0500-7-226)
Supplement: Additional file 2: — Experimental Overview, part 2. Description; Contains an overview of the microarray experiment for the last five biological replicates of samples. [file 1756-0500-7-226-S2.pdf]

Microarray data for The Commonwealth Medical College

Michael Bordonaro, Ph.D.

January 27, 2012

20 Human cell samples
(24 samples used for data analysis)

Summary of procedures for microarray processing

RNA was extracted and purified using Ribopure (Ambion) RNA isolation

Total RNA samples were quantitated by UV spectrophotometry (OD260/280)

Quality of Total RNA was assessed using an Agilent Bioanalyzer

First and second strand cDNA was prepared from the total RNA samples

cRNA target was prepared from the DNA template and verified on the Bioanalyzer

cRNA was fragmented to uniform size and hybridized to Agilent Human v2 GE 4x44K arrays

Slides were washed and scanned on an Agilent G2565 Microarray Scanner

Data was analyzed with Agilent Feature Extraction and GeneSpring GX v7.3.1 software packages

Total RNA quality analysis

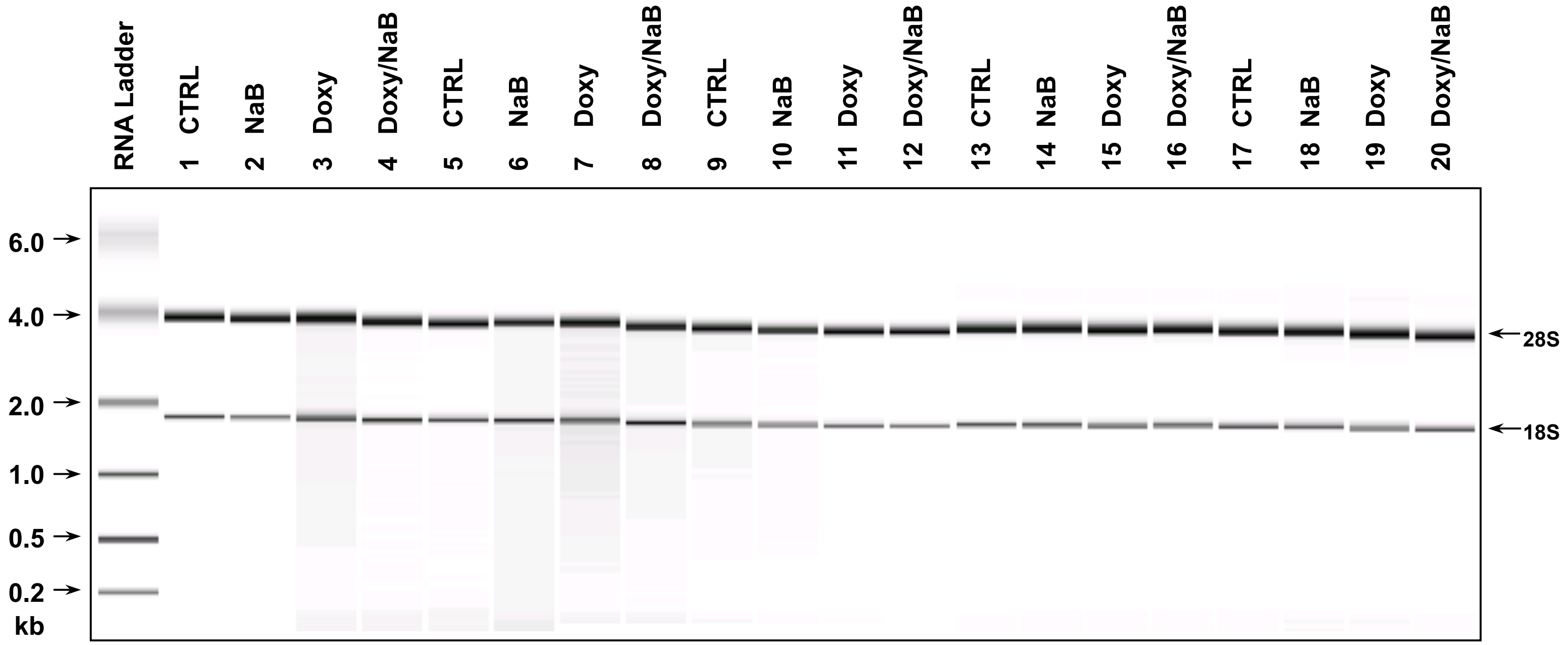

cRNA quality analysis

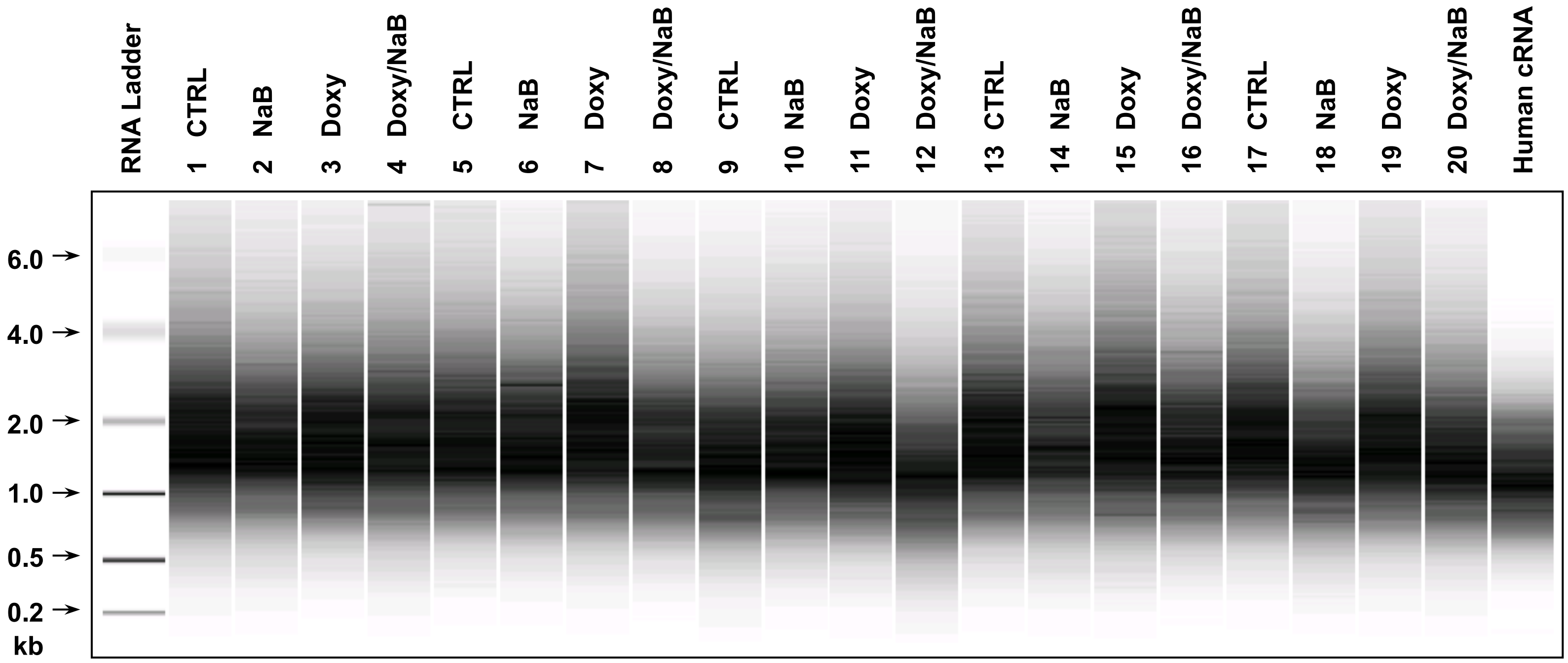

cRNA quality analysis

cRNA electropherograms

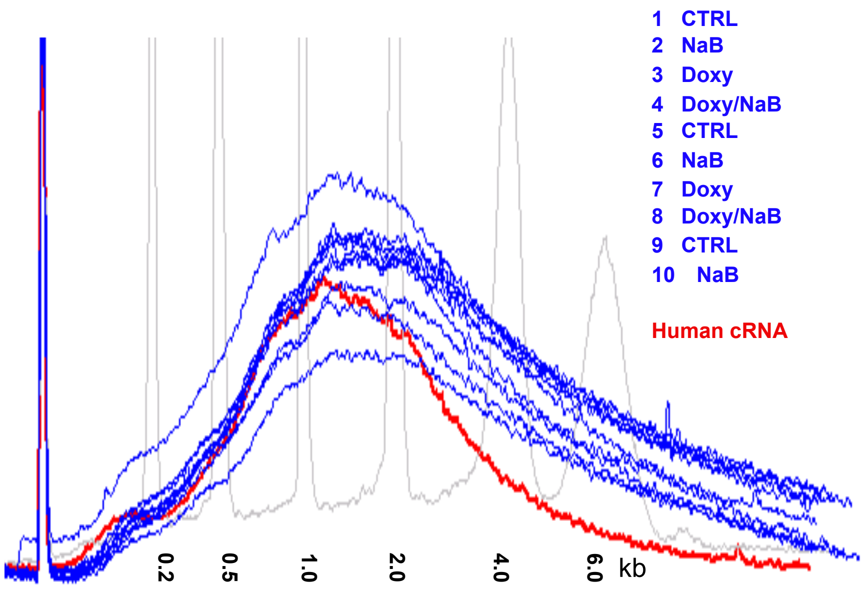

cRNA electropherograms

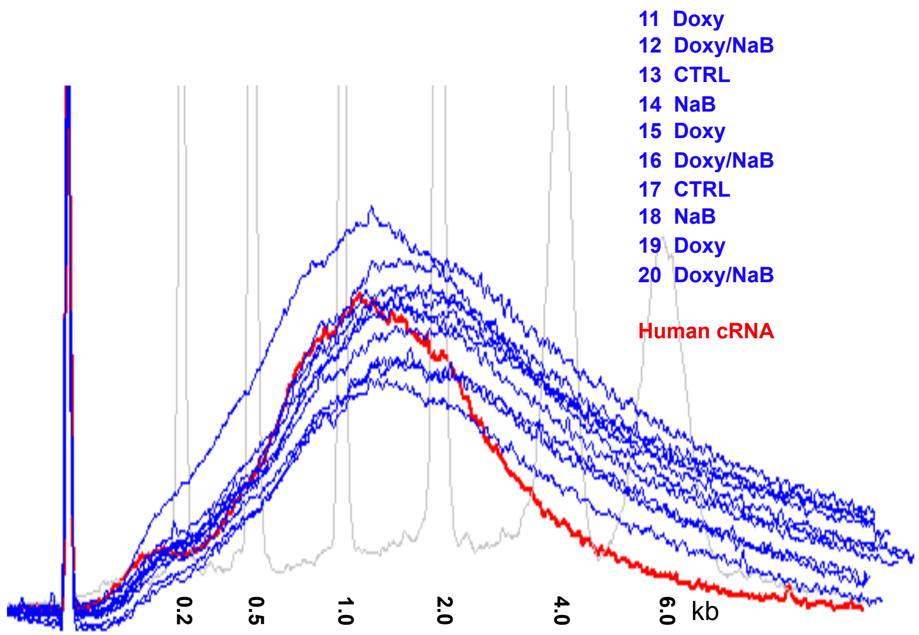

Scatterplot analysis

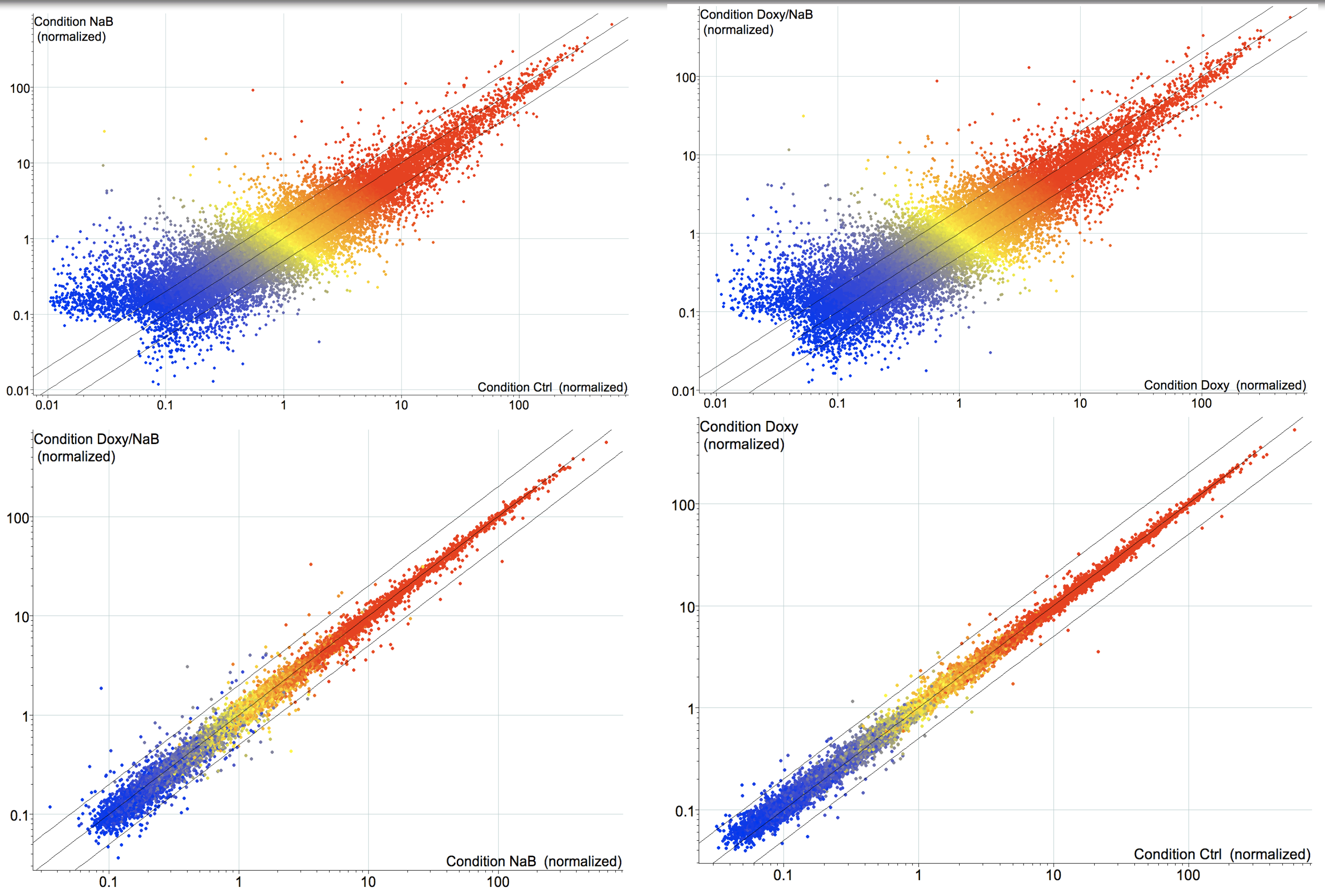

Probes above background in all replicates of either treatment for each comparison (17,847 – 18,779 probes). Intensity values are normalized to the 75th percentile intensity of each array. Diagonal lines indicate 2-fold differential expression. Red/Orange=High expression, Yellow=Medium expression, Blue=Low expression

Condition and Gene tree clustering

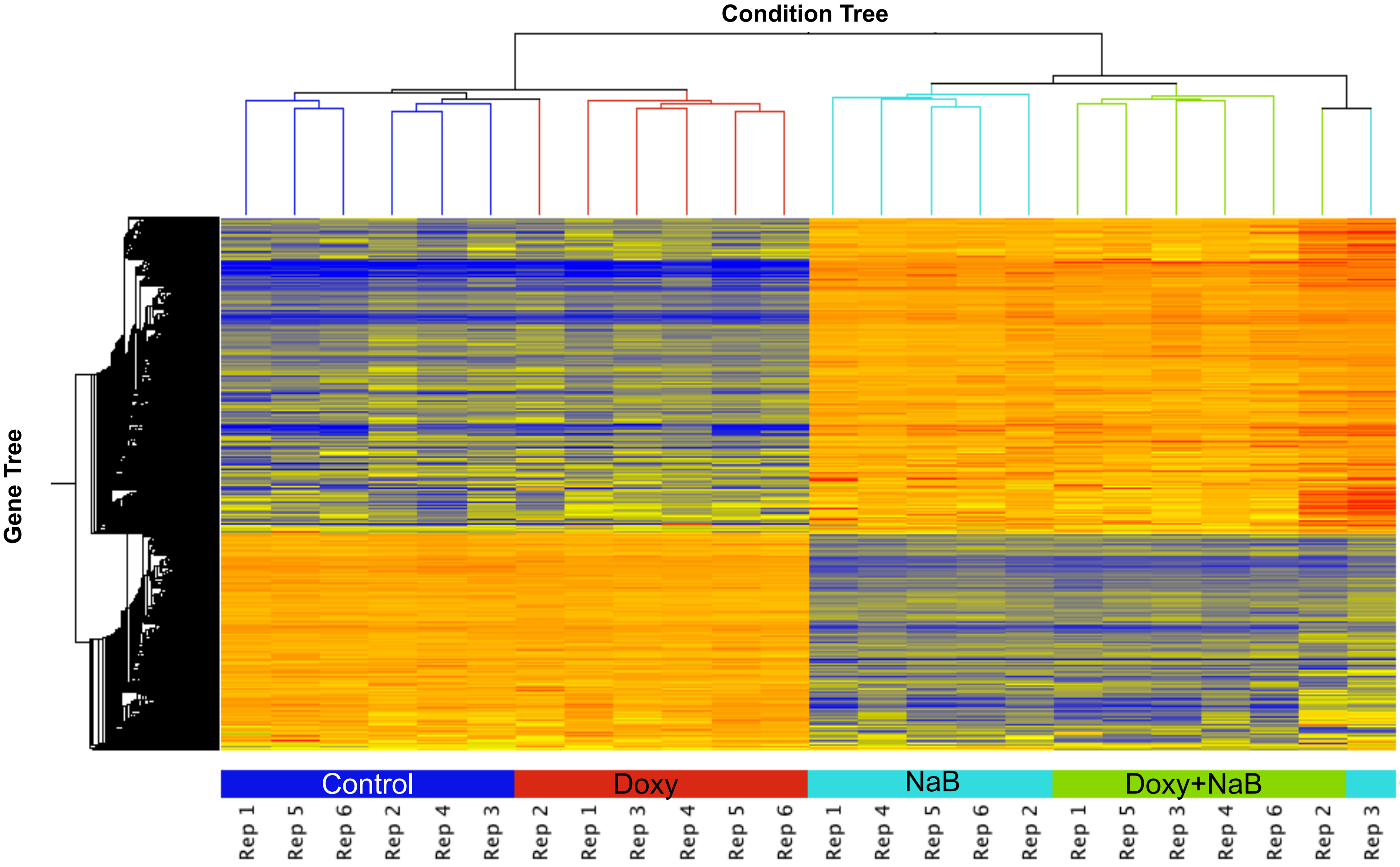

Differentially expressed genes in at least one of four comparisons are displayed as normalized to the median expression across 4 samples within each experiment (>2-fold, paired t-test p-value < 0.01, 6,607 probes). Red/Orange=Up-regulated relative to median, Yellow=Median expression, Blue=Dn-regulated relative to median.

Condition and Gene tree clustering

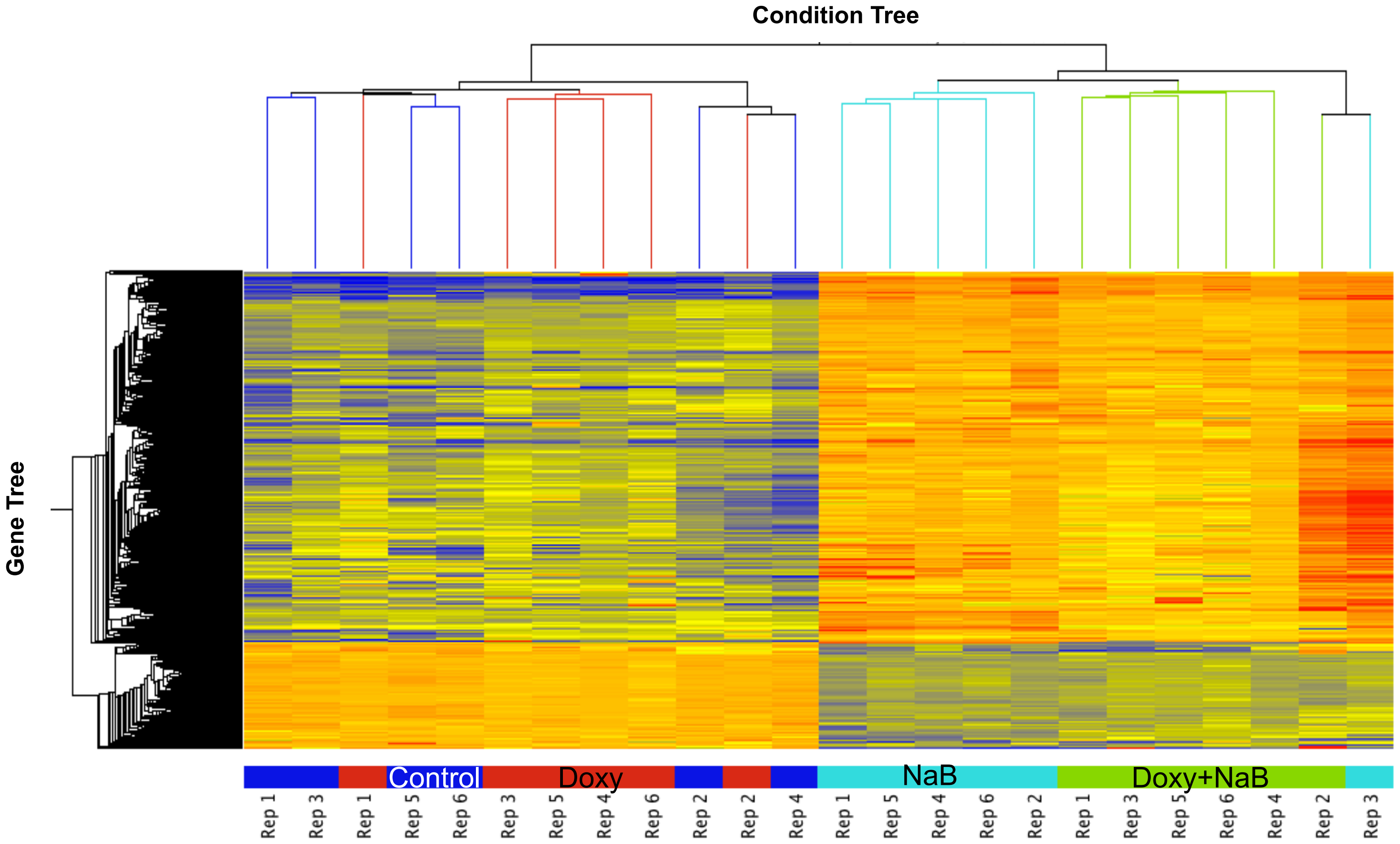

Differentially expressed genes in NaB vs. Control NOT Doxy+NaB vs. Doxy are displayed as normalized to the median expression across 4 samples within each experiment (>2-fold, paired t-test p-value < 0.01, 1008 probes). Red/Orange=Up-regulated relative to median, Yellow=Median expression, Blue=Dn-regulated relative to median.

Condition and Gene tree clustering

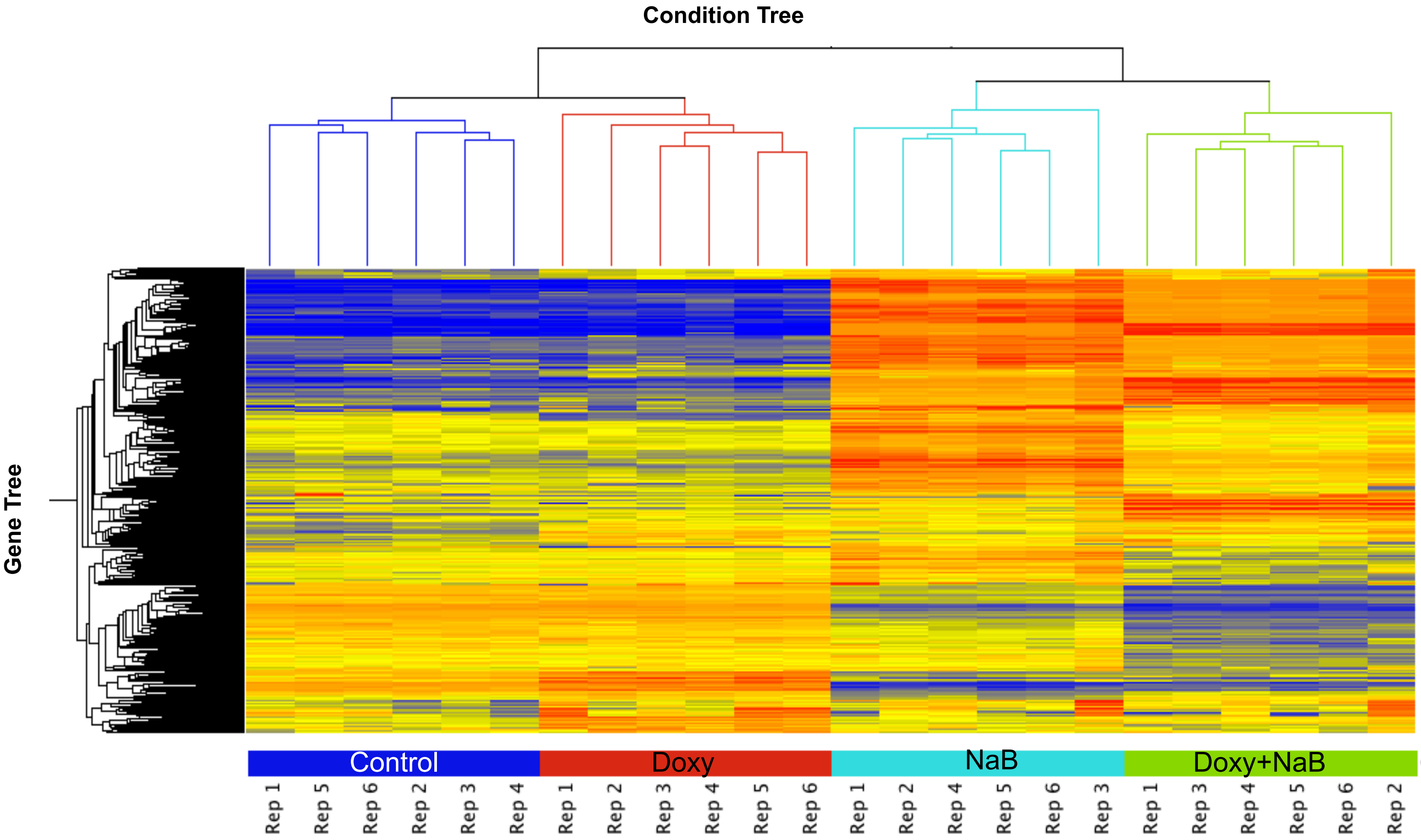

Differentially expressed genes in at least one of two secondary comparisons (Doxy vs. Ctrl or Doxy+NaB vs. NaB) are displayed as normalized to the median expression across 4 samples within each experiment (>1.5-fold, paired t-test p-value < 0.01, 364 probes). Red/Orange=Up-regulated relative to median, Yellow=Median expression, Blue=Dn-regulated relative to median.

GENUS BIOSYSTEMS

Condition and Gene tree clustering

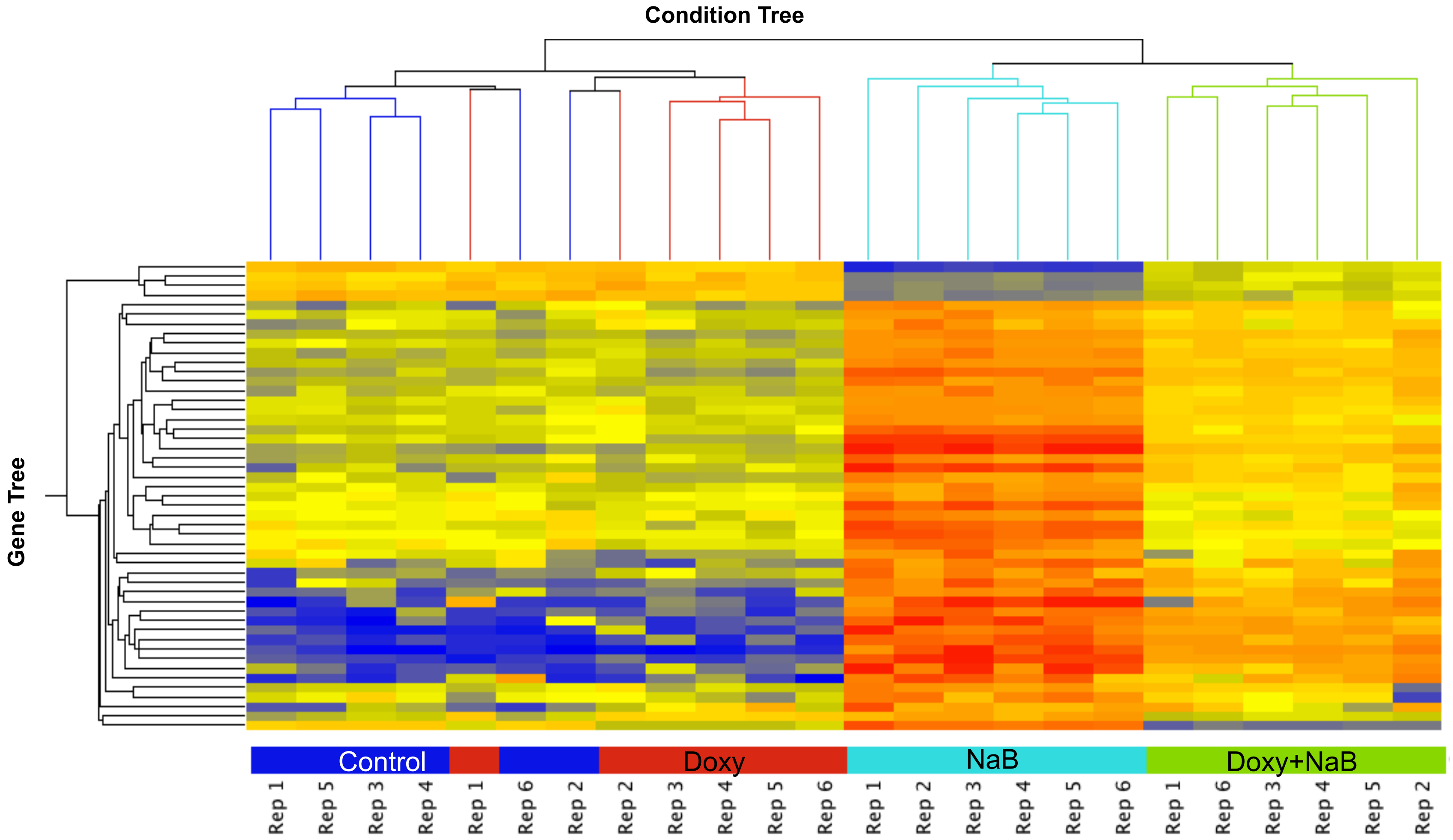

Differentially expressed genes in NaB vs. Control NOT Doxy+NaB vs. Doxy and also different in Doxy+NaB vs. NaB are displayed as normalized to the median expression across 4 samples within each experiment (>2-fold, paired t-test p-value < 0.01, 49 probes). Red/Orange=Up-regulated relative to median, Yellow=Median expression, Blue=Dn-regulated relative to median.

Differentially Expressed Genes

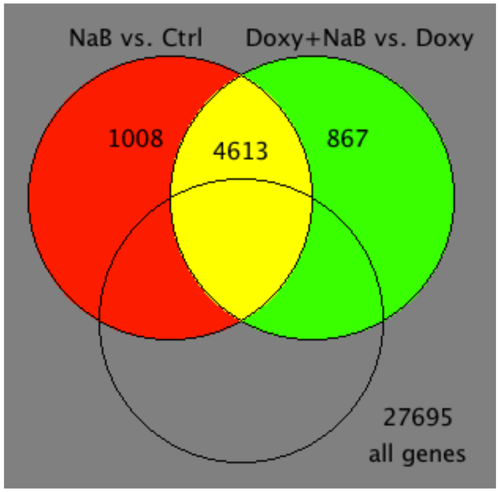

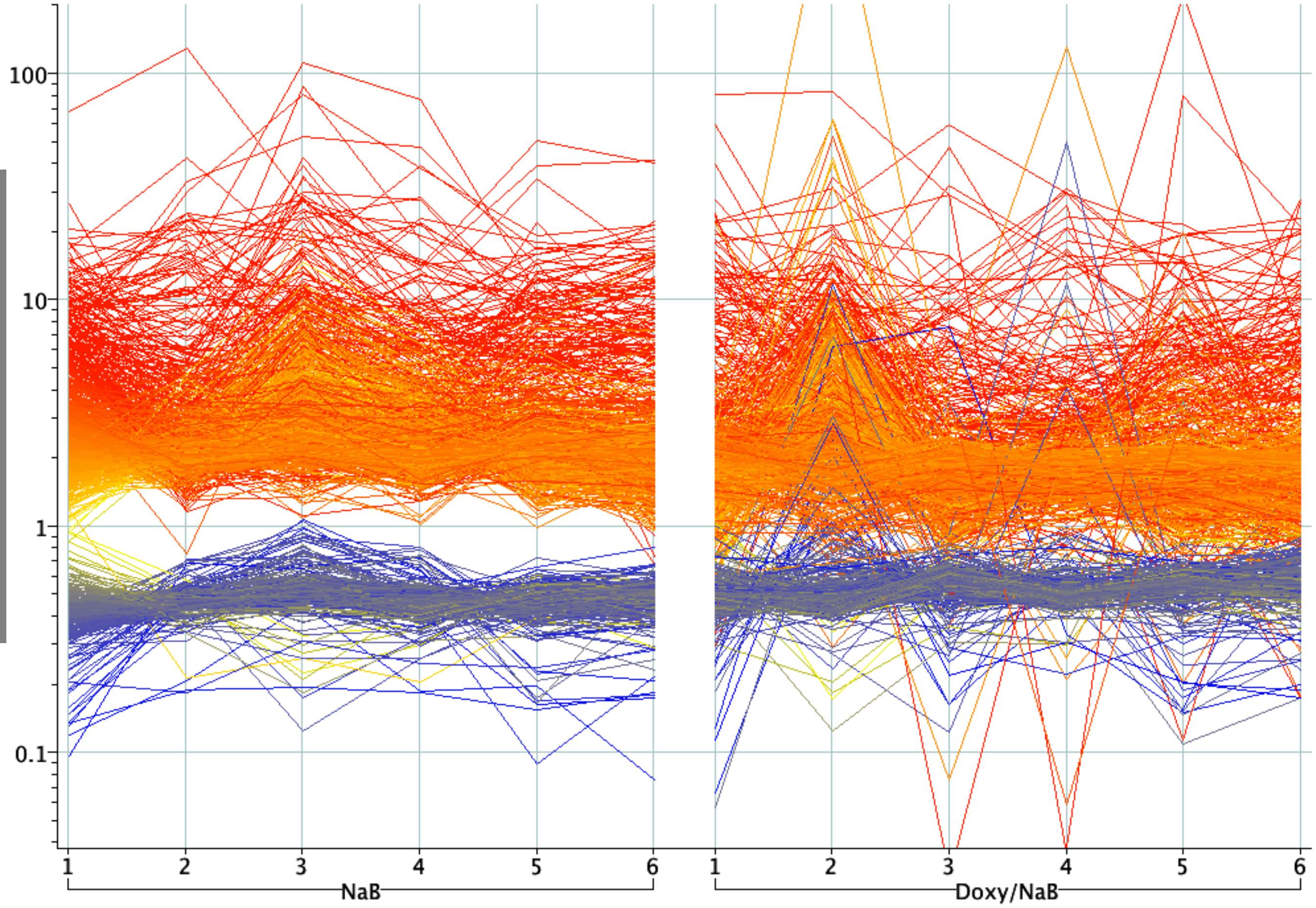

Venn diagram comparison of differentially expressed genes in NaB vs. Ctrl or Doxy+NaB vs. Doxy (>2Fold, Paired T-test p-values < 0.01, 6,488 probes). Only the 1,008 red genes are displayed in the line graph as normalized to the 75th percentile intensity of each array and further normalized to the control or doxy sample for each experiment. Genes are colored by the expression values in the NaB 1 sample. Red/Orange=Up-regulated relative to Ctrl, Blue/Grey=Dn-regulated relative to Ctrl.
